# Anisotropic swelling wound dressings with vertically aligned water absorptive particles†

**DOI:** 10.1039/c7ra13764h

**Published:** 2018-02-21

**Authors:** Yuanhao Guo, Shuyang Pan, Fanhui Jiang, Enmin Wang, Liliana Miinea, Nancy Marchant, Mukerrem Cakmak

**Affiliations:** Department of Polymer Engineering, University of Akron Akron Ohio 44325 USA; Lubrizol Advanced Materials, Inc. Ohio 44092 USA; School of Materials Engineering, School of Mechanical Engineering and Birck Nanotechnology Center, Purdue University West Lafayette IN 47907 USA cakmak@purdue.edu

## Abstract

A multi-layer solution casting method was utilized to fabricate a three-layer wound dressing film consisting of a wound contact layer, an absorbing layer and a backing layer. The absorbing layer, whose function is to absorb and retain the exudate thus providing a moist environment for wound healing, was made of superabsorbent particles and a thermoplastic polyurethane matrix. In this study, the superabsorbent particles were aligned into chains whose axes oriented along the thickness direction of the film by an external electric field. This structure could minimize the lateral swelling of the absorbing layer while preferentially expanding in the thickness direction during the water absorption process, and therefore eliminate the lateral stress or shear induced friction between the films and the wound. When compared to the wound dressing films with non-aligned particles, the films with aligned particles could achieve up to 33% smaller lateral expansion. The effect of particle shape on anisotropic swelling was also investigated, and the rod-like particles with higher aspect ratio were more effective at improving the anisotropic swelling and reducing lateral expansion compared to irregular-shaped particles. Additionally, the imprinted patterns on the contact layer resulting from the electric field alignment process promoted the efficiency of absorbing and transporting the exudate into the absorbing layer.

## Abstract 2

An anisotropic swelling multi-layer wound dressing with vertically orientated water absorptive particles induced by an electric-field.

## Graphical abstract

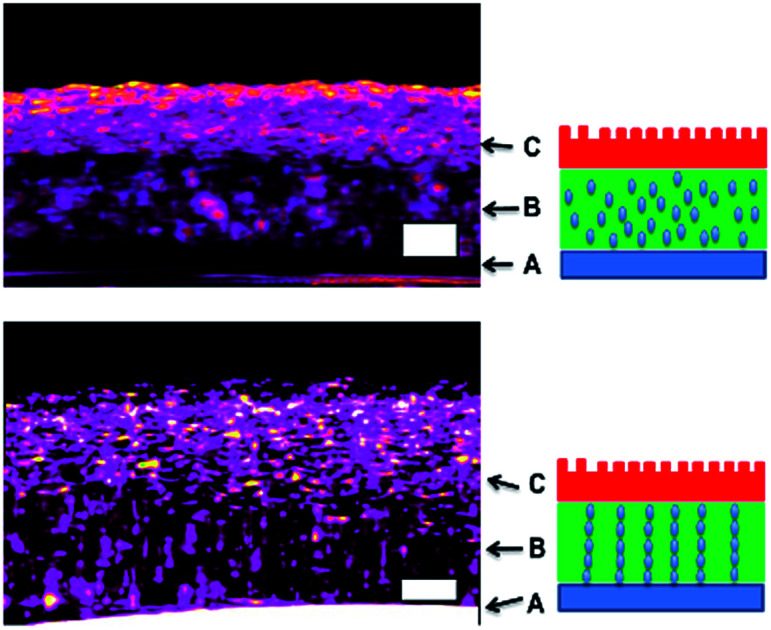

## 1. Introduction

Wound healing is a very complex biochemical and cellular process, which comprises the five overlapping stages of haemostasis, inflammation, migration, proliferation and maturation.^1,2^ Once the haemostasis stage is achieved, exudate, essentially blood free of red cells and platelets, is produced in both acute and chronic wounds.^3,4^ The exudate plays an important role for wound healing by keeping a moist environment^5^ and supplying nutrients, mitosis of epithelial cells and leucocytes to aid the wound healing as well as to protect the wound from infections.^6^ In some severe cases such as deep acute wounds and chronic wounds, excess amount of exudate is produced, which inhibits the wound healing and corrupts the healthy skin around the wound due to the high level of proteinase enzymes.^7,8^ Traditional wound dressings such as cotton wool, natural bandage, lint and gauze were commonly used for wound care in the past.^9^ These traditional wound dressings could absorb the exudate and prevent the wound from infection by bacteria, but they failed to restrain the evaporation of moisture, which resulted in the wound becoming overly dried. Furthermore, their adhesion to the wound could cause overwhelming discomfort and pain, or even more tissue damage during the removal of the wound dressings. Recent research has found that a warm and moist wound environment can help accelerate healing.^1,10^ Therefore, polymer-based wound dressings that are able to absorb and retain the excess exudate, hence maintaining moist environment and showing no or less adhesion to the wound are desirable.^9^

A number of polymer-based wound dressings have been developed to promote the wound healing and may be classified into: hydrocolloid dressing,^11,12^ alginate dressing,^13^ hydrogel dressing^14,15^ and collagen dressing^16^ based on the materials used to fabricate them. Among them, hydrocolloid dressing, which is a semi-permeable polyurethane film with absorbent particles^9,17^ is the most widely used, ascribed to the fact that the absorbent particles inside provide excellent exudate absorption capacity, and become gels after absorption to induce fast healing rate and less pain.^18,19^ In addition to absorption capacity, the ability to retain the moisture is also crucial for wound dressings. Nevertheless, traditional single layer wound dressings were insufficient to withhold the absorbed moisture due to the high moisture vapour transportation rate (MVTR), which could cause the desiccation of wound.^20^ Therefore, multilayer wound dressings that consist of a semipermeable backing layer, an absorbent layer and a wound contact layer have been developed to absorb and also to retain the exudate, benefiting from a better control on the MVTR of the backing layer.^20,21^ One of the major drawbacks of the multilayer wound dressings is that the highly absorptive layer undergoes a large lateral expansion after absorbing the exudate, which may cause lateral shearing or friction stress to the wound. This stress hinders the wound healing and may cause further sores or damage the extremely fragile periwound area.^21^

To solve this problem, many efforts have been made to develop anisotropic polymer films that would absorb fluid and expand in the vertical direction. T. Kajiyama *et al.*^22^ fabricated stearyl acrylate (SA)/acrylic acid (AA) copolymer gel film with the lamellar layer aligning parallel to film surface and alkyl chains oriented in thickness direction (“*Z*” direction). When such films were immersed in ethanol, their swelling ratio in “*Z*” direction was 40% larger than in-plane direction due to the anisotropic crystal structure. M. Sisido *et al.*^23^ prepared a polymer gel of nematic liquid crystalline (NLC) poly(γ-benzyl l-glutamate) (PBLG) with its helix axis oriented along the surface of film by external magnetic field,^24^ the swelling ratio of which in “*Z*” direction was two times that of in-plane swelling ratio in dichloroacetic acid. This was attributed to the fact that the mechanism of swelling in NLC state was the insertion of solvent between the parallel helix, which mainly contributed to vertical expansion.^23^ However, the films in the above studies didn't show anisotropic swelling behavior in water. To achieve anisotropic swelling in water, G. Sigurjonsson^25^ reported a multilayer wound dressing with a specially processed absorbent layer: the absorbent core was drilled with sub millimeter sized cylindrical receptacles vertically in a predetermined pattern, and these cylindrical compartments were filled with superabsorbent. The wound contacting layer was also drilled with large number of apertures so that the absorbent layer could attract the exudate due to capillary action. Then the vertically aligned superabsorbent cylinders would absorb the exudate and expand to push the backing layer in the thickness direction. However, the preparation procedures in this method involve drilling receptacle, filling superabsorbent and laminating three layers, which are too complicated. The size of the vertical superabsorbent cylinder is too large, in the range of sub millimeter causing non-uniformity issues.

In the present study, a multi-layer solution casting method was utilized to fabricate the three-layer wound dressing films consisting of a wound contact layer, an absorbing layer and a backing layer. The absorbing layer, whose function is to absorb and retain moisture, was made of superabsorbent particles dispersed in a thermoplastic polyurethane matrix. The rod-like or irregular shape superabsorbent particles were prepared by cryogrinding and electrospinning methods, respectively. The water absorptive particles were aligned *in situ* in the presence of electric field in “*Z*” direction to form vertical micro or nano size particle chains or cylinders in the absorbent layer as opposed to filling the submillimeter size superabsorbent material in a complex process as the one described by G. Sigurjonsson.^25^ The effect of particle shape and alignment on anisotropic swelling behavior was also studied.

## 2. Results and discussions

**Fig. 1:**
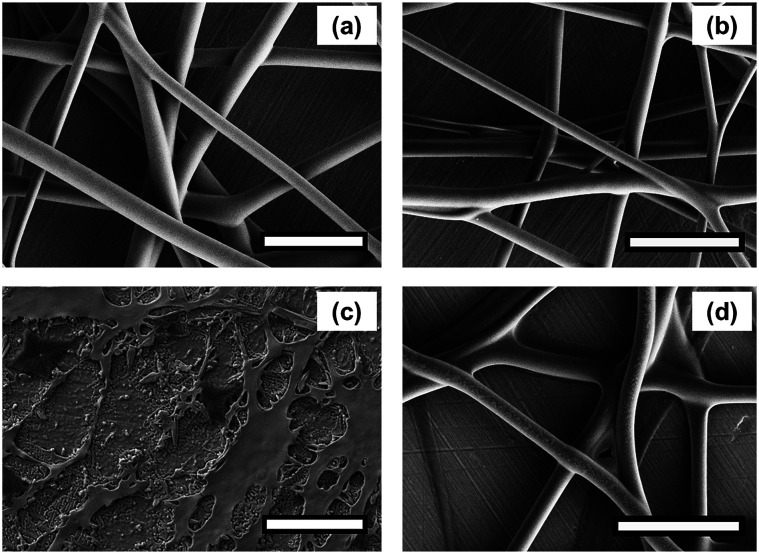
SEM morphology of PAA-1-Na/PEG nanofibers (a) as-spun, (b) after cross-linking, (c) nanofibers without crosslinking after washing, (d) cross-linked nanofiber after washing (scale bar: 5 μm).

The three-layer wound dressing films prepared in this study consist of a wound contact layer, an absorbing layer and a backing layer. The absorbing layer was made of superabsorbent particles and a thermoplastic polyurethane matrix. The superabsorbent particles were prepared by neutralizing the polyacrylic acid polymers with sodium hydroxide aqueous solution (18 wt%) to pH 7 in order to improve their exudate absorbing ability. The superabsorbent particles were also crosslinked to maintain their integrity after absorbing exudate. The two materials used for preparing the rod-like (nanorods) and irregular-shaped superabsorbent particles in this study were based on polyacrylic acid polymers PAA-1 and PAA-2, respectively. Nanorods were prepared by electrospinning the aqueous solution of PAA-1 neutralized to pH 7 and PEG, followed by mechanically grinding resulting nanofiber mat. Fig. 1a shows the SEM morphology of the as-spun PAA-1-Na/PEG nanofibers with an average diameter of 700 nm. Since PAA-1 used for electrospinning was a linear and non-crosslinked polymer, the as-prepared PAA-1-Na/PEG nanofibers were crosslinked afterwards at 190 °C *via* the esterification reaction between the carboxyl group of PAA-1 and the hydroxyl group of PEG to avoid their dissolution in water. The morphology of PAA-1-Na/PEG nanofibers remained the same after thermal induced crosslinking shown in Fig. 1b. To confirm the crosslinking of nanofibers, the as-spun and crosslinked nanofiber mats were immersed in solution A for 30 minutes. As can be observed in Fig. 1c, the as-spun PAA-1-Na/PEG nanofibers dissolved and spread into films, whereas the crosslinked nanofibers mostly maintained their integrity despite some fibers slightly clumping together with each other as shown in Fig. 1d.

**Fig. 2:**
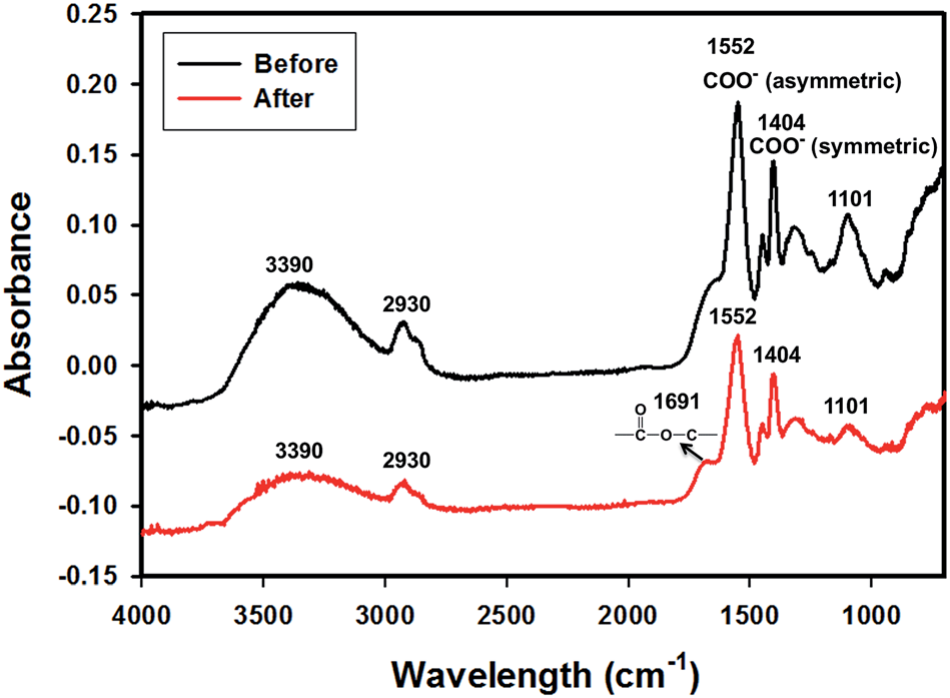
FTIR of neutralized PAA-1-Na/PEG nanofibers before and after crosslinking.

**Fig. 3:**
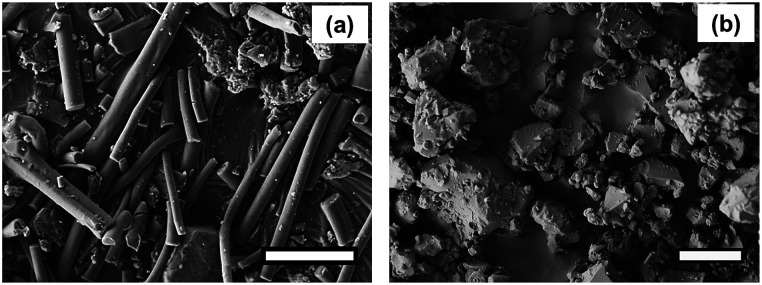
SEM morphology: (a) PAA-1-Na/PEG nanorods (scale bar: 5 μm), (b) PAA-2-Na irregular-shaped particles (scale bar: 10 μm).

In order to obtain evidence of crosslinking, the FTIR spectra of the PAA-1-Na/PEG nanofibers were recorded before and after reaction as shown in Fig. 2. The absorption at 3390 cm^−1^, 2930 cm^−1^, 1450 cm^−1^ and 1101 cm^−1^ were due to the stretching vibration of O–H, C–H, CH_2_ and C–O groups, respectively.^26^ The peaks at 1552 cm^−1^ and 1404 cm^−1^ were assigned to asymmetric and symmetric stretching of –COO^−^, respectively,^27,28^ because the PAA-1 was neutralized by sodium hydroxide solution. The area decrease of these two peaks was due to the conversion of the ionic bonds to ester bonds (–CO_2_C–).^29^ Meanwhile, the absorption band at 1691 cm^−1^ corresponding to carboxylic ester group appears after crosslinking. The morphology of the PAA-1-Na/PEG nanorods prepared by mechanically grinding the nanofiber is shown in Fig. 3a. The cross-linked PAA-2 was used to prepare the irregular particles by neutralization and cryogrinding (Fig. 3b).

**Fig. 4:**
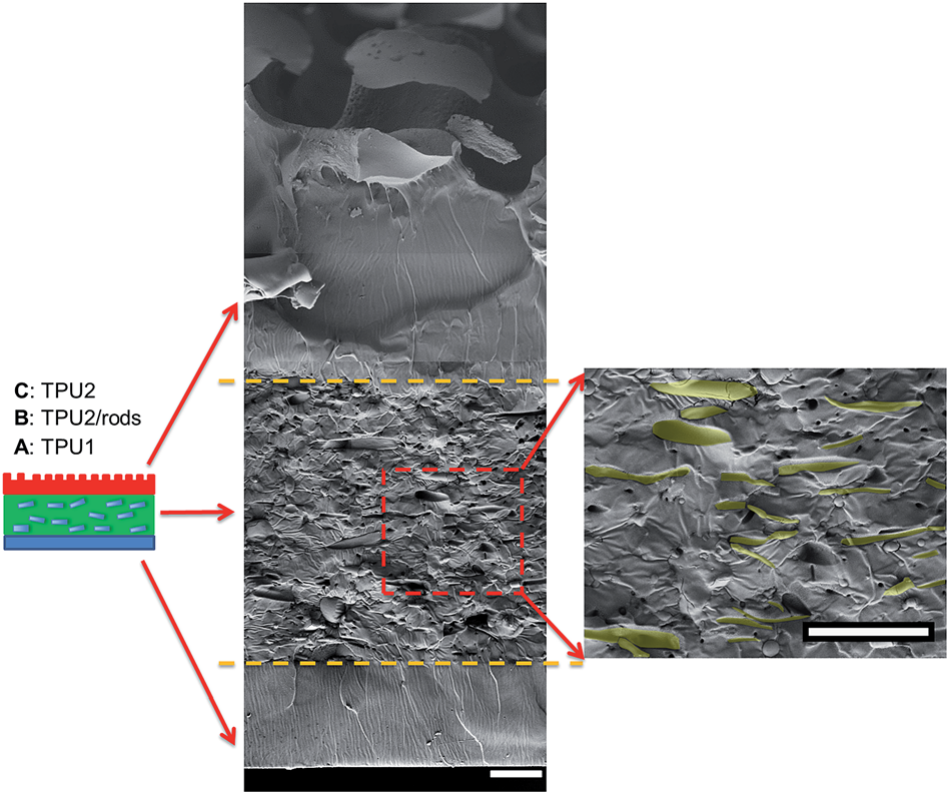
Cross sectional morphology of the three-layer wound dressing film without application of electric field (nanorods were artificially colored to enhance the contrast) (scale bar: 20 μm).

**Fig. 5:**
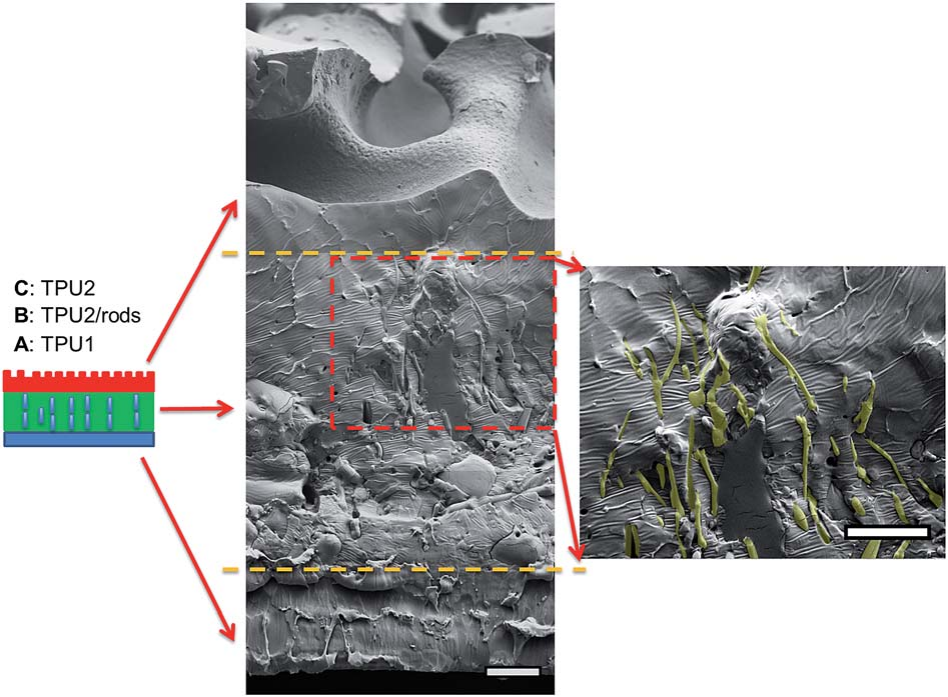
Cross sectional morphology of the three-layer wound dressing film with aligned nanorods (nanorods were artificially colored to enhance the contrast) (scale bar: 20 μm).

The three-layer wound dressing films were prepared by solution casting method, and the *in situ* alignment of nanorods or irregular particles was achieved by applying electric field in “*Z*” direction of film until the film was dried. The orientation and alignment of nanorods in TPU2/dioxane solution was observed by optical microscope and recorded in ESI Video 1.† SEM was utilized to characterize the cross sectional morphology of the resulting three-layer wound dressing films as shown in Fig. 4. This figure illustrates the control morphology of the three-layer film without the application of electric field. A clear three-layer structure could be seen: layer A, layer B with dispersed nanorods, and layer C with imprinted patterns. Layer A was made of semipermeable polyurethane (TPU1), which acted as a backing layer to control the MVTR. Layer B was composed of super absorbent PAA-1-Na/PEG nanorods and polyurethane matrix (TPU2). The nanorods were colored yellow (false color) to enhance the contrast between matrix and dispersed particles. For the three-layer wound dressing films obtained without applying electric field, the nanorods were all oriented parallel to the surface of film due to development of compression force caused by the thickness shrinkage as the solvent evaporates.^30^ On the other hand, for the three-layer wound dressing films prepared with the application of electric field, the nanorods were oriented and aligned in thickness direction as shown in Fig. 5. This is attributed to the fact that the dielectrophoretic force overcomes the compression force during the drying resulting in maintenance of the developed orientation of the long axes of the particles in thickness direction.^31,32^ Some of the nanorods were not oriented perfectly in “*Z*” direction and a small tilting angle was observed between the electric field direction and the long axis of nanofibers due to the shrinkage stress as the film thickness reduces,^31^ but overall a clear orientation and alignment of the nanorods were obtained along the film thickness direction. Layer C is a polyurethane layer (TPU2) with imprinted patterns replicating the feature of Teflon coated mesh.

**Fig. 6:**
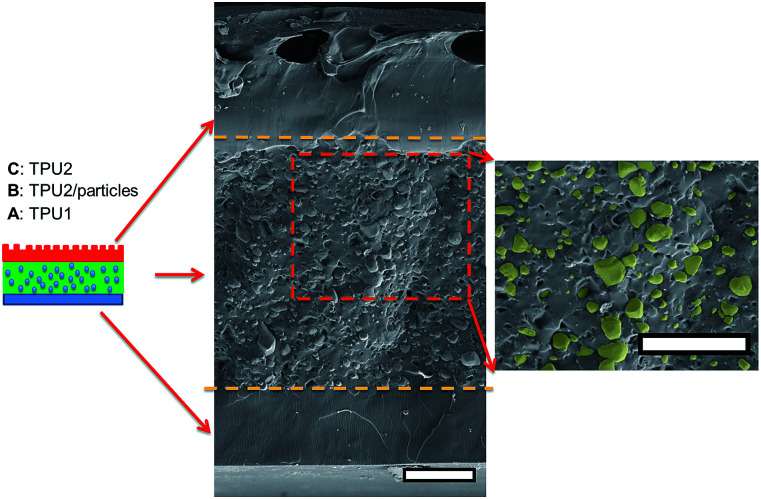
Cross sectional morphology of the three-layer wound dressing film with random irregular-shaped particles (scale bar: 40 μm).

**Fig. 7:**
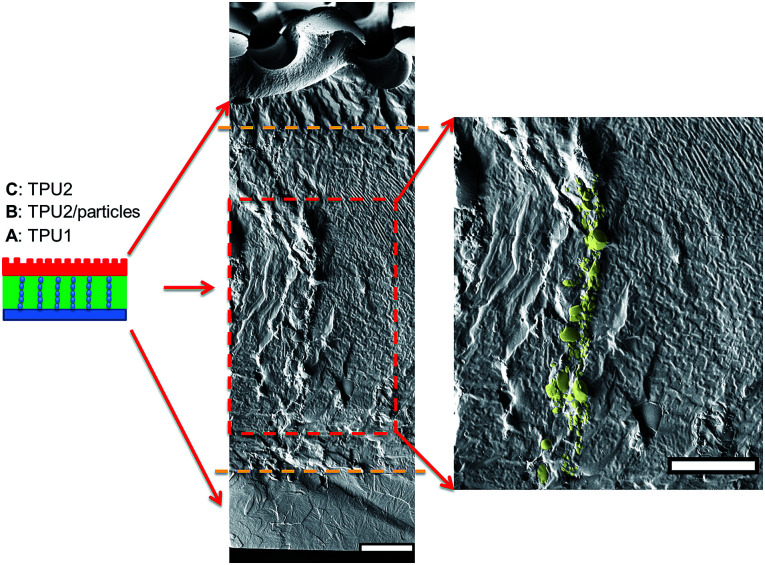
Cross sectional morphology of the three-layer wound dressing film with aligned irregular-shaped particles (scale bar: 40 μm).

**Fig. 8:**
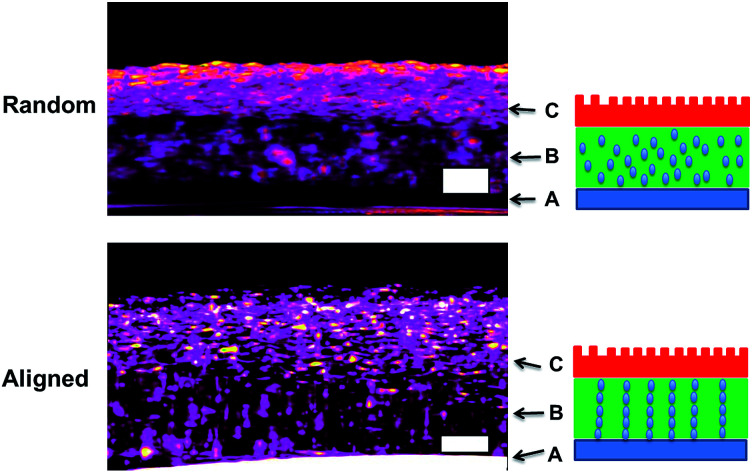
Micro-CT morphology of three-layer wound dressing films with random and aligned irregular-shaped particles (scale bar: 100 μm).

As for irregular particles, the cross-section of resulting three-layer wound dressing films were also characterized by SEM for both non-aligned and aligned samples, illustrated in Fig. 6 and 7, respectively. Similar to nanorods, irregular particles were aligned in the film thickness direction, and formed into chain structures in layer B for the samples with the application of electric field, whereas the irregular particles were randomly distributed in the films prepared without applying electric field. For a direct visual observation, the video showing the alignment of irregular particles in TPU/dioxane solutions was provided in ESI Video 2.† Additionally, the non-aligned and aligned samples were also examined by the micro-X-ray tomography system to obtain the three dimensional distribution of irregular particles, as shown in Fig. 8. For the three-layer wound dressing film prepared with no electric field, irregular-shaped particles were randomly distributed in layer B, whereas they were assembled into chains in the three-layer wound dressing film prepared with electric field. Such three-layer films, when used in wound dressing applications, were flipped upside down. Namely, the layer C with imprinted patterns becomes the layer that directly contacts the wound and transports the exudate towards layer B. Layer B is the absorbing layer which absorbs and retains the exudate. Layer A is the backing layer that allows the moisture to evaporate through but prevent ingress of liquid from outside.

**Fig. 9:**
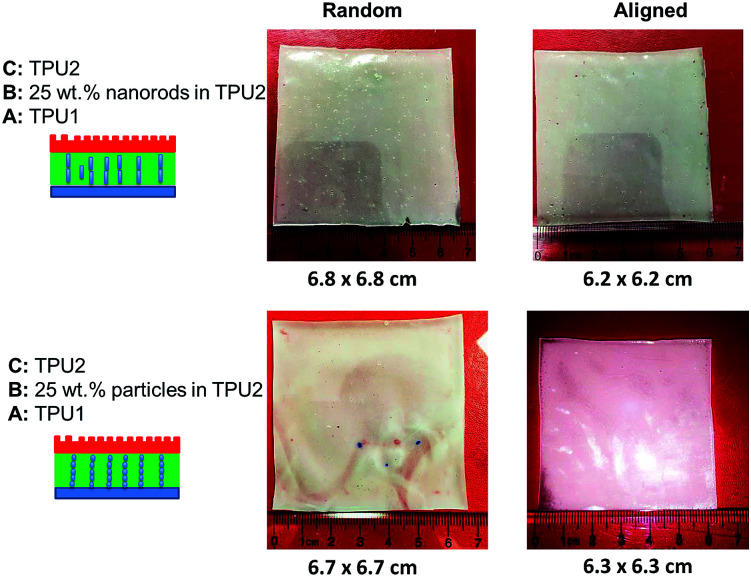
The size comparison of wound dressing films with random and aligned nanorods or irregular-shaped particles after swelling.

**Fig. 10:**
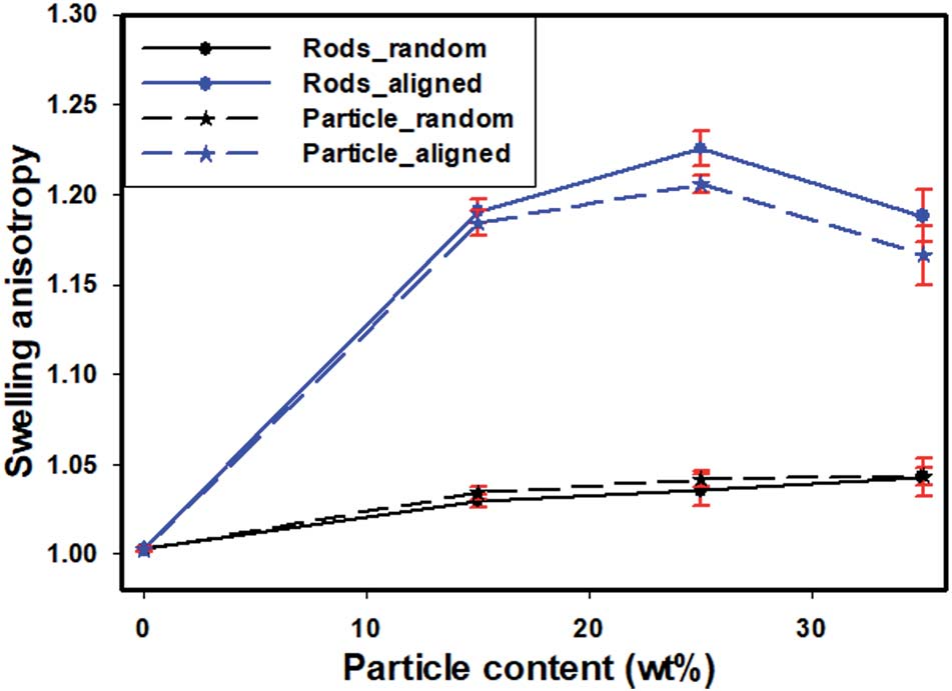
The effect of alignment and particle shape on swelling anisotropy as a function of particle content.

To study the swelling behavior of resulting films, the three-layer wound dressing films with a series of particle contents (0, 15, 25 and 35 wt%) were cut into 5 × 5 cm size and soaked in solution A at 37 °C for 30 minutes. All the swollen samples were measured and samples with 25 wt% particles are shown in Fig. 9 as representatives. The three layer wound dressing films with non-aligned and aligned nanorods expanded from 5 × 5 cm to 6.8 × 6.8 cm and 6.2 × 6.2 cm, respectively. The lateral expansion was reduced by 33% after alignment indicating that directional swelling is actually working in these films. Since the nanorods attained much lower modulus than the polyurethane matrix in the gel state after absorbing water, the stiffer matrix that surrounds the aligned nanorod chains restrains the expansion in the lateral direction, leading to more vertical deformation. Similar effects could also be seen for irregular particles. However, less significant reduction in the lateral expansion was obtained for films with aligned irregular particles, due to their lower aspect ratio and larger particle sizes. The in-plane dimension of all swollen samples with various particle concentrations (0, 15, 25 and 35 wt%) and the sample pictures were provided in ESI (Table S1, Fig. S1 and S2†). To quantitatively evaluate the anisotropic swelling properties of each sample, the swelling anisotropy value was calculated as the ratio of out-of-plane expansion ratio (*R*_⊥_) over in-plane expansion (*R*_∥_), and plotted as a function of particle concentration in Fig. 10. Generally, the three-layer wound dressing films with aligned nanorods or irregular particles achieved dramatically higher swelling anisotropy values than those with randomly dispersed particles, meaning that the aligned wound dressing films underwent more vertical expansion than less lateral expansion in comparison to non-aligned films. Also, the three-layer wound dressing films with nanorods exhibited slightly higher anisotropy value than those containing irregular particles due to their higher aspect ratios. There're much fewer numbers of particles needed to form chains with nanorods than irregular particles, so there's less sliding among the nanorods as they expand after absorbing the exudate. The swelling anisotropy values of the three-layer wound dressing films with aligned nanorods or irregular particles all showed first increasing then decreasing trend as the particle content increased from 0 to 35 wt%, and the maximum swelling anisotropy value was achieved at 25 wt%. The swelling ratio of the three-layer wound dressing films increases with particle content as expected in Fig. S3(a).† The MVTR of the three-layer wound dressing films with various particle contents was characterized and plotted in Fig. S3(b).† The three-layer wound dressing films with nanorods or irregular particles demonstrated virtually identical MVTR values between aligned and non-aligned samples, which all located between 700 and 1600 g m^2^ 24 h^−1^. The wound dressings with three-layer structures were in the desired range of MVTR for ideal wound dressings to maintain optimal moist environment to avoid frequently wound dressing replacement^20^ The true stress-true strain curve of the swollen multi-layer wound dressing film is shown in Fig. S4† with a Young's modulus of 5.2 MPa, so it is mechanically strong enough as a wound dressing.

**Fig. 11:**
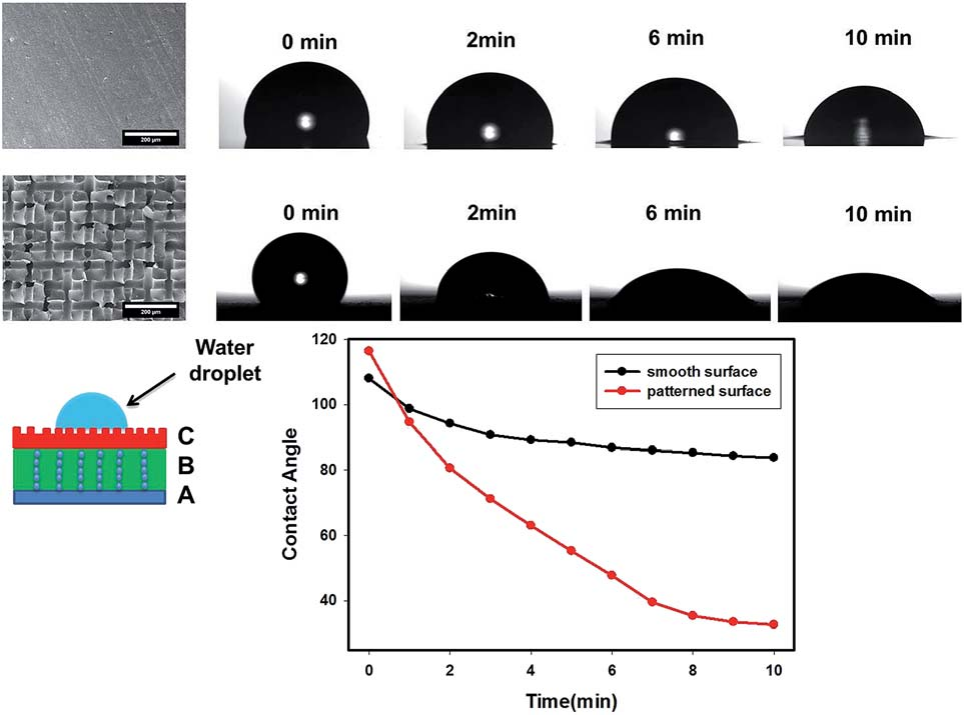
The effect of surface of pattern of layer C on the contact angle as a function of time.

As mentioned earlier, the top surface layer C of the three-layer wound films were imprinted by the conductive mesh electrode pattern shown in Fig. 11. Since layer C was the contact layer that absorbed the excess exudate directly from the wound, it was expected to be hydrophilic and wettable for moisture to transport through. The effect of these imprinted patterns on the wettability of wound dressing films was evaluated by measuring the contact angle of water droplet on layer C, and compared to the three-layer films with smooth surface prepared by the same procedures but without the use of top electrode (mesh). The contact angle values were plotted as a function of wetting time for both smooth and patterned surface films in Fig. 11. The contact angle on the patterned surface started with slightly higher value (116°) than smooth surface films (107°), due to their higher roughness level as predicted by the Wenzel model. This model predicts that the contact angle of this material with rough surface increases with the roughness if the contact angle of this material with smooth surface is above 90°.^33,34^ However, the contact angle of the three-layer wound dressing films with patterned surface almost instantly dropped below that of smooth surface films and reached a much lower value of 32° at 10 min than 83° of smooth surface films. This is attributed to the capillary force and higher surface area induced by the patterned surface. Moreover, a higher base radius of the water droplet on the patterned surface was observed, which is consistent with previous study.^35^ A combination of lower contact angle and higher base radius of water droplet indicates that the film with patterned surface has better water absorbing ability, which is an additional benefit except direction swelling from the electric field alignment process.

## 3. Conclusions

Three-layer wound dressing films with anisotropic swelling were prepared by preferentially aligning the absorbent particles along film thickness direction in the absorbent layer. The resulting wound dressings exhibited greater vertical expansion than lateral expansion after absorbing water owing to the formation of columnar organization particles in the thickness direction. This is extremely beneficial in reducing the shear stress on the wound. The particle shape played an important role in the anisotropic swelling behavior of aligned wound dressing films. It was found that cylindrical particles (nanorods), which possessed higher aspect ratio, could further increase the anisotropic swelling ratio and reduce the lateral expansion than irregular-shaped particles. Another advantageous effect was that the top layer with imprinted patterns imposed by the porous electrode achieved lower contact angle as well as higher base radius of the water droplet. The three-layer wound dressing films exhibit MVTR values between 700 and 1600 g m^2^ 24 h^−1^, demonstrating enough breathability to maintain a balanced moist wound environment that accelerate wound healing and avoid maceration.

## 4. Experimental section

### 4.1 Material

The water absorptive polymers, Carbopol® 981NF and Carbopol® 907, were supplied by the Lubrizol Corporation and used to prepare water absorptive particles. Thermoplastic polyurethanes (TPU): Tecophilic™ SP80A150 and Tecophilic™ HP60D20, were also provided by the Lubrizol Corporation. 1,4-Dioxane (ACS reagent, ≥99.0%), *N*,*N*-dimethylacetamide (DMAc) (ACS reagent, ≥99%), polyethylene glycol (PEG) (Mn = 400 g mol^−1^), sodium chloride (ACS reagent, ≥99%), calcium chloride dihydrate (ACS reagent, ≥99%) and sodium hydroxide (NaOH) (reagent grade, ≥98%, pellets) were purchased from Sigma Aldrich. Teflon coated stainless steel mesh obtained from TWP. Inc. was used as the top electrode. Indium Tin Oxide (ITO) coated glass supplied by Structure Probe, Inc. was used as the bottom electrode. The alternating current (AC) voltage was generated by high voltage (HV) amplifier (AMP-20B20, Matsuda Precision Inc.). Solution A having ionic composition comparable to wound dressing, was prepared by dissolving 8.298 grams of sodium chloride and 0.368 grams of calcium chloride dihydrate in 1 liter of deionized water.

### 4.2 Preparation of superabsorbent nanorods

12 wt% Carbopol® 907 (non-crosslinked polyacrylic acid (PAA-1)) aqueous solution was prepared by using a paddle mixer. The resulted solution was neutralized with sodium hydroxide aqueous solution (18 wt%) to pH 7 and 3 wt% PEG with respect to Carbopol® 907 was added into the above solution to act as the crosslinker. The resulted mixture was electrospun into nanofibers (PAA-1-Na/PEG) by using electrospinning technique in the following conditions: 25 gauge needle, 22 kV voltage and 13 cm target distance. The collected PAA-1-Na/PEG nanofiber mat was kept in oven at 190 °C for 7 min to drive the esterification reaction between polyacrylic acid and PEG. The cross-linked PAA-1-Na/PEG nanofiber mat was placed in a container with ceramic balls and grinded into PAA-1-Na/PEG nanorods by ball grinding in Thinky mixer at 2000 rpm for 2 minutes.

### 4.3 Preparation of superabsorbent irregular particles

1 wt% Carbopol® 981NF (crosslinked polyacrylic acid (PAA-2)) aqueous dispersion was prepared by using a paddle mixer, and was neutralized with sodium hydroxide aqueous solution (18 wt%) to pH 7. The resulting viscous dispersion was cast into 3 mm thick film and dried at 80 °C overnight. The dried film was grinded into large irregular particles (PAA-2-Na) in Thinky mixer at 2000 rpm for 5 minutes. The irregular particles were further grinded into smaller PAA-2-Na irregular particles by cryogrinding.

### 4.4 Preparation of the three-layer wound dressing film

**Fig. 12:**
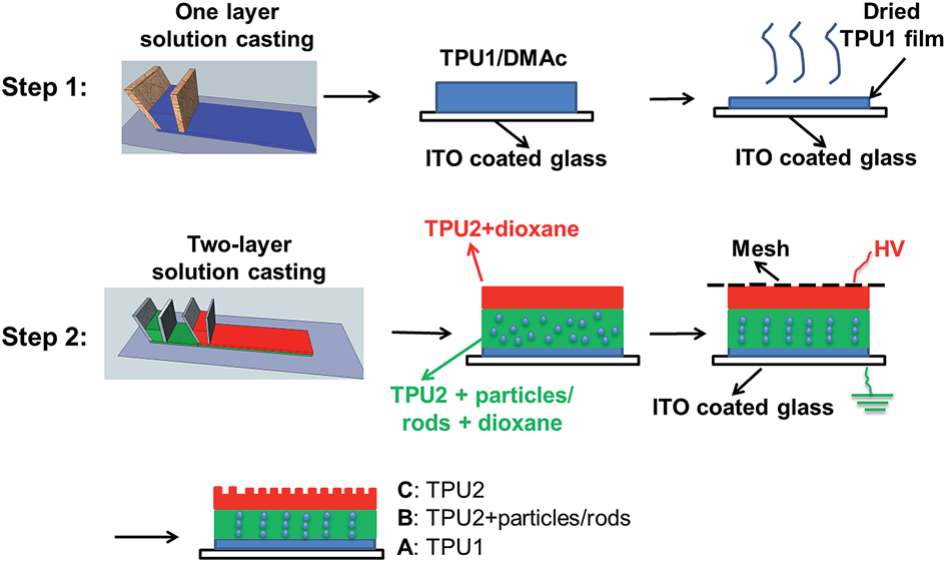
Procedures to prepare the three-layer wound dressing films.

The two-step preparation procedures were used to prepare the three-layer wound dressing films as shown in Fig. 12. The solution of 15 wt% Tecophilic™ HP60D20 in DMAc was cast on ITO coated glass (bottom electrode) at 0.5 mm thickness by doctor blade and dried at 50 °C for 12 hours, and this layer of film was marked as layer “A”. Herein, Tecophilic™ HP60D20 and Tecophilic™ SP80A150 were denoted as TPU1 and TPU2, respectively. In the second step, the two-layer solution casting method was used to cast the other two-layer solution films on the top of dried TPU1 film. The solution used for middle layer preparation was a 19 wt% TPU2 in dioxane solution with various concentrations of neutralized nanorods or irregular particles. The solution used for the preparation of the top layer was a 19 wt% TPU2 in dioxane solution. The resulted three-layer solution film was covered by a Teflon coated mesh (top electrode).^31^ 600 V mm^−1^ (AC) initial electric field was applied between mesh and ITO glass for 6 hours until all the solvent evaporated. The middle and top layer of dried film was marked as layer “B” and layer “C”, respectively.

The three-layer wound dressing films with non-aligned nanorods or irregular particles were prepared following the same procedures above except that no electric field was applied between the mesh and ITO glass during drying.

### 4.5 Morphology characterization

The morphology of PAA nanofibers and nanorods was characterized by JEOL-7401 scanning electron microscopy (SEM). The random and aligned three-layer films were fractured in the liquid nitrogen, and the cross sectional morphology was also studied by SEM. All the samples were sputter coated with silver prior to observation. The 3D morphology of random and aligned wound dressing film was characterized by Bruker Skyscan 1172 micro-computed tomography (micro-CT).

### 4.6 Swelling test

The three-layer wound dressing films prepared above were cut into 5.0 × 5.0 cm square shaped samples and soaked in the solution A for 30 min at 37 °C according to British Standard BS EN 13726-2:2002. Five parallel samples were repeated for each sample. The weight, thickness and length before and after swelling were measured to calculate the swelling ratio (SR), in plane expansion ratio (*R*_∥_), out of plane expansion ratio (*R*_⊥_) and swelling anisotropy (SA) in equations below:1
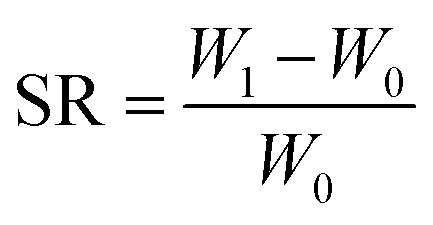
2
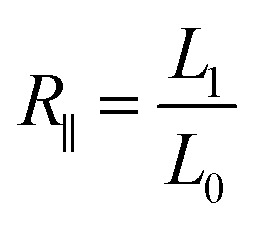
3
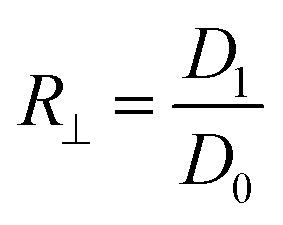
4
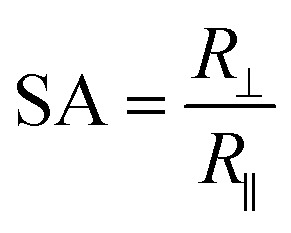
where *W*, *L* and *D* is weight, length and thickness of the three-layer film, respectively. 0 and 1 represent before and after swelling, respectively. *R*_∥_ and *R*_⊥_ are in plane and out of plane expansion ratio, respectively.

### 4.7 Moisture vapor transmission rate (MVTR)

The three-layer wound dressing films were sealed on the top of a cup with inner diameter 35.7 mm containing 20 ml solution A. The cup was kept in an incubator with a temperature of 37 °C and humidity of less than 20% and the MVTR is calculated according to British Standard BS EN 13726-2:2002 as follow:5
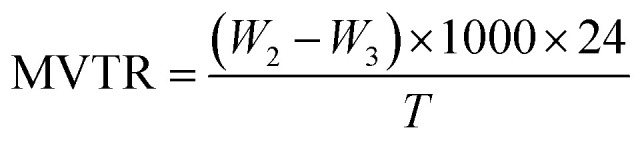
where *W*_2_ and *W*_3_ are the mass of the cup, film and solution A before and after test respectively, and *T* is the test period in hours.

### 4.8 Contact angle of three-layer wound dressing films

The contact angle of the layer C surface of three-layer wound dressing film was studied by DSA 100. The contact angle image was taken every 1 minute after water droplet was on the surface of the film with smooth or patterned surface.

## Conflicts of interest

There are no conflicts of interest to declare.

## Supplementary Material

RA-008-C7RA13764H-s001

RA-008-C7RA13764H-s002

RA-008-C7RA13764H-s003
