# Supplementary material for: Anisotropic swelling wound dressings with vertically aligned water absorptive particles
Source: RSC Adv. 2018 Feb 21;8(15):8173–80. doi: 10.1039/c7ra13764h (PMC9078563; doi:10.1039/c7ra13764h)
Supplement: RA-008-C7RA13764H-s003 [file RA-008-C7RA13764H-s003.pdf]

Electronic Supplementary Material (ESI) for RSC Advances.
This journal is © The Royal Society of Chemistry 2018

Supporting Information

Anisotropic Swelling Wound Dressings with Vertically Aligned Water Absorptive Particles

Yuanhao Guo[a], Shuyang Pan[a], Fanhui Jiang[a], Enmin Wang[a], Liliana Miinea[b], Nancy Marchant[b], Mukerrem Cakmak[a,c,]*

[a] Department of Polymer Engineering, University of Akron, Akron, Ohio 44325, USA
[b] Lubrizol Advanced Materials, Inc. Ohio, 44092, USA
[c] School of Materials Engineering, School of Mechanical Engineering and Birck Nanotechnology Center, Purdue University, West Lafayette, IN, 47907, USA
E-mail: cakmak@purdue.edu

Table S1. The effect of alignment on the lateral expansion on the three-layer wound dressing films after swelling.

Particle content (wt.%)	Size of the films with nanorods (cm)		Size of the films with irregular-shaped particles (cm)	
	Random	Aligned	Random	Aligned
0	5.93	5.91	5.92	5.93
15	6.65	6.06	6.56	6.12
25	6.87	6.23	6.78	6.32
35	7.06	6.54	7.04	6.71

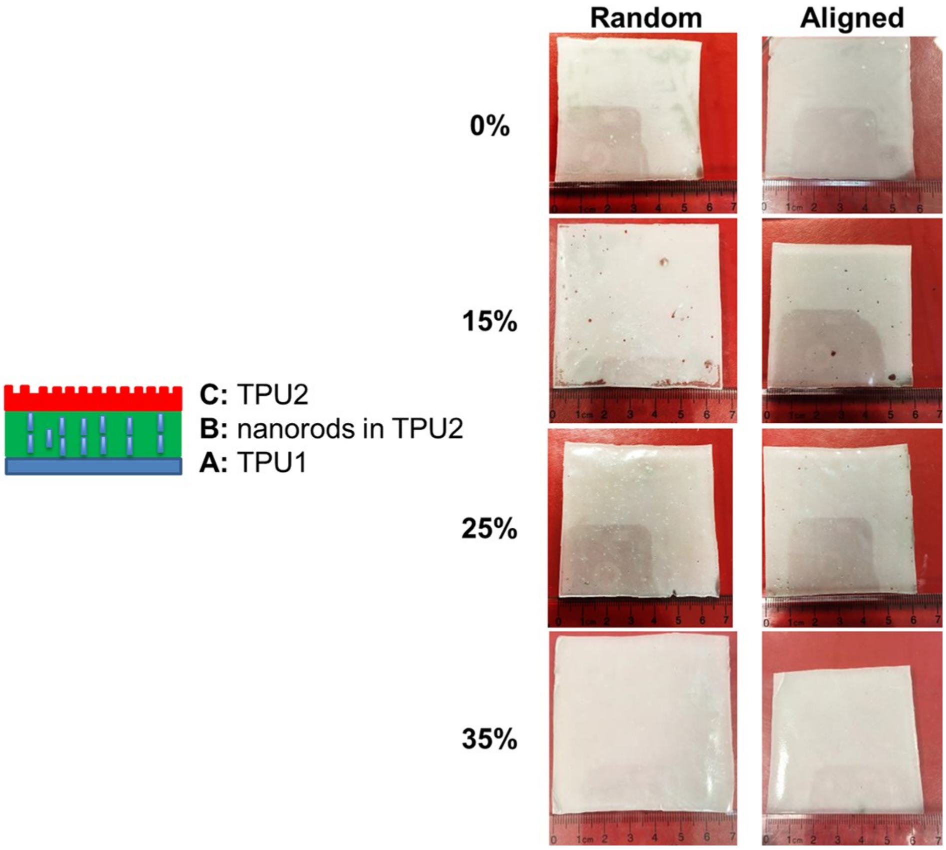

Figure S1. The effect of alignment of nanorods on the lateral expansion on the three-layer wound dressing films with nanorods after swelling.

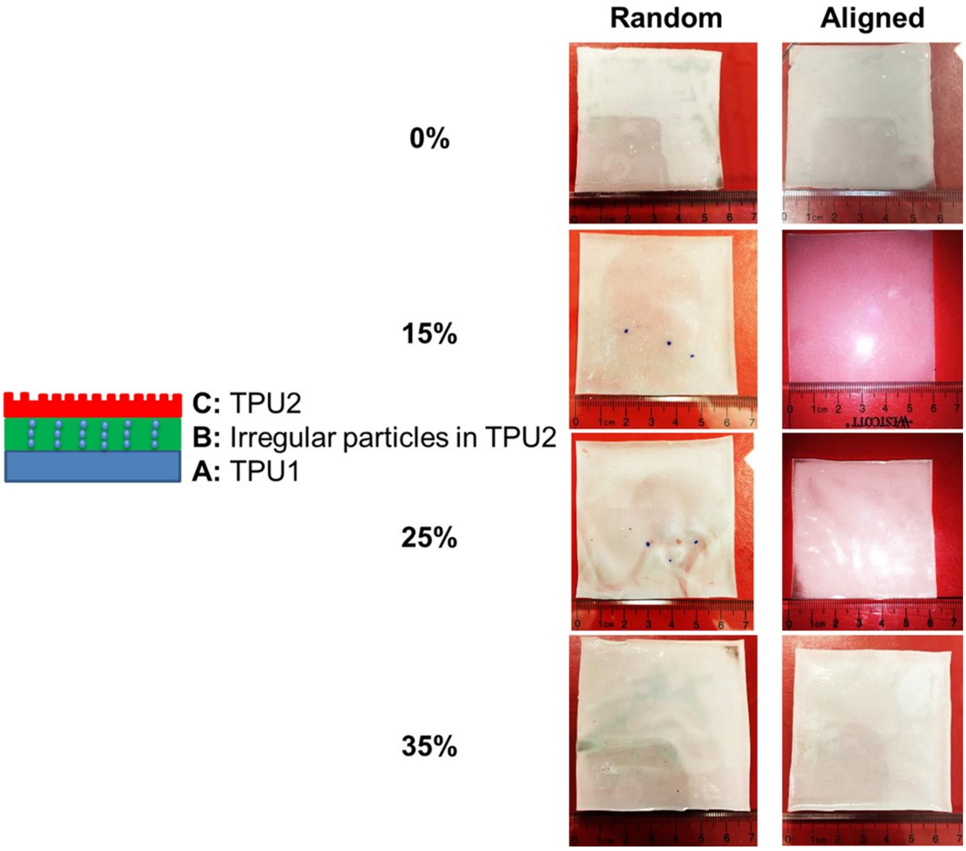

Figure S2. The effect of alignment on the lateral expansion on the three-layer wound dressing films with irregular-shaped particles after swelling.

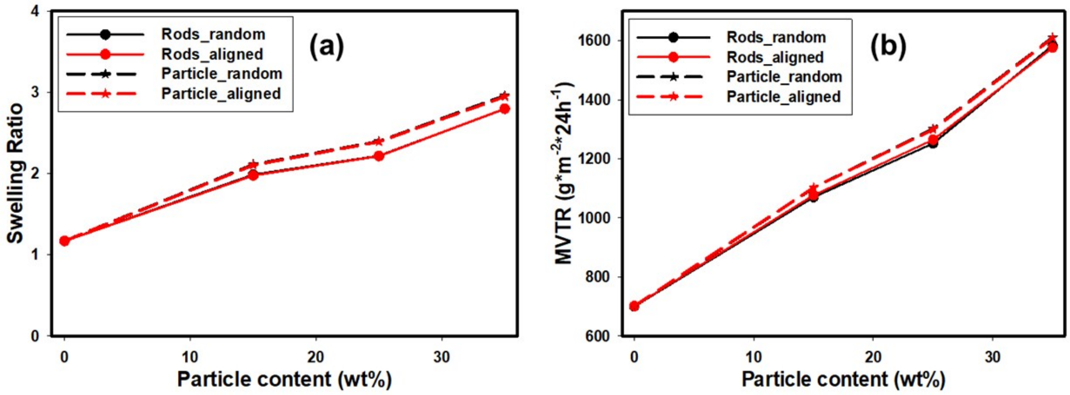

Figure S3. The effect of alignment on (a) swelling ratio and (b) MVTR of three-layer wound dressing films after swelling.

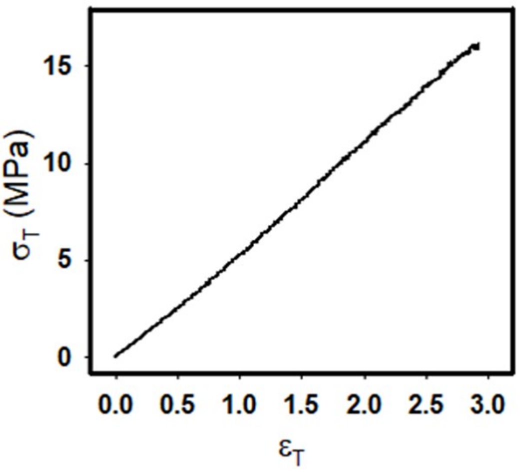

Figure S4. True stress verse true strain curve of the swollen multi-layer wound dressing films.

Video S1: Electric field induced alignment of nanorods in 19 wt.% TPU2 in dioxane solution under 600 V/mm (scale bar: 50 μm).

Video S2: Electric field induced alignment of irregular-shaped particles in 19 wt.% TPU2 in dioxane solution under 600 V/mm (scale bar: 50 μm).
